# TNM stage I pancreatic ductal adenocarcinoma patients without pathological risk may drive limited benefit from adjuvant chemotherapy

**DOI:** 10.3389/fmed.2026.1914610

**Published:** 2026-09-14

**Authors:** Zhifei Li, Xiaoxu Chen, Yuexiang Liang, Batu Bao, Xidong Wang, Meng Zhang, Chuntao Gao

**Affiliations:** 1 Department of Hepatobiliary-Pancreatic-Splenic Surgery, Inner Mongolia Autonomous Region People’s Hospital, Hohhot, China; 2 Inner Mongolia Clinical Medical College, Inner Mongolia Medical University, Hohhot, China; 3 Department of Pancreatic Cancer, National Clinical Research Center for Cancer, Key Laboratory of Cancer Prevention and Therapy, Tianjin, Tianjin’s Clinical Research Center for Cancer, Tianjin Medical University Cancer Institute & Hospital, Tianjin, China

**Keywords:** adjuvant chemotherapy, pancreatic ductal adenocarcinoma, pathological risk, stage I, TNM

## Abstract

**Objective:**

In this study, we identified pathological risk factors as key determinants of whether patients with pancreatic ductal adenocarcinoma (PDAC) can benefit from adjuvant chemotherapy following radical resection.

**Methods:**

This single-center retrospective study enrolled 520 patients who underwent curative-intent resection for pancreatic ductal adenocarcinoma between 2011 and 2018. To minimize potential selection bias and confounding effects, propensity score matching (PSM) was performed using a 1:2 nearest-neighbor matching algorithm without replacement, with a caliper of 0.2 standard deviations of the logit propensity score. After matching, the patients were stratified based on the presence or absence of pathological risk factors, allowing for a comprehensive evaluation of how these factors influence the efficacy of adjuvant chemotherapy.

**Results:**

Based on the analysis for the whole study, the results showed that adjuvant chemotherapy could significantly improve the overall survival (OS) (*p* < 0.001) and recurrence-free survival (RFS) (*p* < 0.001). However, for patients in Tumor-Node-Metastasis (TNM) stage I without pathological risk factors, postoperative adjuvant chemotherapy did not significantly prolong the OS (median OS: NA vs.50.0 months, *p* = 0.365) and RFS (median RFS: 24 months vs.44 months, *p* = 0.569); for patients with pathological risk factors, adjuvant chemotherapy can significantly improve the OS (median OS: 28.0 months vs.17.0 months, *p* < 0.001) and RFS (median RFS: 21.0 months vs.7.0 months, *p* = 0.004).

**Conclusion:**

The results of this study indicate that pathological risk factors play a key role in postoperative adjuvant chemotherapy for PDAC patients in TNM stage I. For patients in TNM stage I who harbored pathological risk factors, postoperative adjuvant chemotherapy was associated with a significant survival benefit. However, among those without pathological risk factors, no statistically significant association was observed between adjuvant chemotherapy and improved survival outcomes.

## Introduction

Following radical resection for pancreatic ductal adenocarcinoma (PDAC), patients are typically recommended to receive comprehensive postoperative adjuvant chemotherapy. This approach aims to eliminate potential residual tumor cells and address occult metastatic lesions that may not be detectable on imaging, ultimately improving overall survival and delaying both tumor recurrence and metastasis (1, 2). Multiple high-quality randomized controlled trials have demonstrated that adjuvant chemotherapy significantly improves the prognosis of patients with PDAC (3–7). However, whether these survival benefits are consistently observed across all TNM stages remains a subject of debate.

Given the low detection rate of early-stage PDAC and the scarcity of evidence supporting adjuvant chemotherapy specifically for TNM stage I disease, most existing studies have predominantly concentrated on patients with TNM stage II—III. To date, no clinical guideline has explicitly recommended postoperative adjuvant therapy for patients with TNM stage I PDAC, as its survival benefit in this population remains insufficiently established (8). Two retrospective studies by Katherine T et al. (9) and Michael Guenther et al. (10) have suggested that postoperative adjuvant chemotherapy confers a significant survival advantage in patients with TNM stage I PDAC, advocating for its routine adoption in this subgroup. In contrast, a recent meta-analysis (8) that such chemotherapy did not significantly improve the 3-year or 5-year survival rates in patients with TNM stage I PDAC. Adding to this uncertainty, a large cohort study of 612 patients with resectable pancreatic cancer demonstrated that gemcitabine-based adjuvant chemotherapy did not significantly improve survival in the subset of patients with N2 lymph node metastasis (11). Therefore, whether postoperative adjuvant chemotherapy confers a meaningful survival advantage in patients with TNM stage I PDAC remains an unresolved question, warranting further investigation.

These findings suggest that relying solely on TNM staging to determine the indication for postoperative adjuvant chemotherapy may be inadequate. A comprehensive assessment integrating specific pathological characteristics is therefore essential for optimizing treatment decisions. In this context, factors such as vascular invasion, perineural infiltration, and lymph node metastasis have been consistently identified as negative prognostic indicators in PDAC and may potentially influence the therapeutic efficacy of adjuvant chemotherapy (12–14). In line with these observations, previous work by our team has further demonstrated that poor tumor differentiation and elevated Ki-67 expression (defined as Ki-67 ≥30%) are independently associated with adverse survival outcomes in patients with PDAC (15). Poor differentiation was selected to represent histological dedifferentiation and invasive potential, while a Ki-67 index >30% was chosen to represent high proliferative activity. These two indicators reflect complementary biological dimensions of tumor aggressiveness. However, whether these pathological factors also modulate the efficacy of adjuvant chemotherapy remains inadequately explored.

In the present study, patients will be stratified into two groups based on the presence or absence of the aforementioned pathological risk factors—namely, poor tumor differentiation and Ki-67 ≥30%, to evaluate whether these features modulate the response to adjuvant chemotherapy. Our primary objective is to elucidate how adjuvant chemotherapy influences patient prognosis across distinct pathological backgrounds, thereby providing a scientific rationale for individualized treatment in PDAC. The results of this investigation are expected to offer valuable guidance for clinicians in selecting patients who are most likely to derive benefit from adjuvant therapy, ultimately facilitating more informed treatment decisions and potentially improving both survival outcomes and quality of life.

## Materials and methods

### Study design and patients

This study was approved by the Ethics Committee of Tianjin Medical University Cancer Institute & Hospital. All procedures were performed in accordance with the ethical standards of the Declaration of Helsinki. Written informed consent was obtained from all patients prior to surgery, authorizing the collection and use of their clinicopathological data and biospecimens for future research purposes. A total of 836 patients who underwent surgical resection for pancreatic cancer at Tianjin Medical University Cancer Institute & Hospital between January 2011 and December 2018 were identified from our institutional database and deemed eligible for this study. The inclusion criteria were as follows: (i) histopathologically confirmed PDAC; (ii) underwent curative-intent R0 resection; (iii) complete clinicopathological data available, including Ki-67 expression status; and (iv) survived the perioperative period. Exclusion criteria comprised: (i) non-PDAC histologies, including adenosquamous carcinoma, acinar cell carcinoma, or carcinomas arising from intraductal papillary mucinous neoplasms or mucinous cystic neoplasms; (ii) bypass surgery or exploratory laparotomy without tumor resection; (iii) R1 or R2 resection; (iv) distant metastasis at presentation; (v) postoperative mortality due to surgical complications; (vi) concurrent or prior malignancy; and (vii) incomplete or missing clinicopathological data.

### Data collection

Clinicopathological data were extracted from our institutional pancreatic cancer database, encompassing the following variables: gender, age at surgery, preoperative serum levels of carbohydrate antigen 19-9 (CA19-9) and carcinoembryonic antigen (CEA), tumor location, surgical procedure, postoperative complications directly related to the operation (including bleeding, anastomotic leakage, pancreatic fistula, chylous fistula, and abdominal or incisional infection), adjuvant chemotherapy status, tumor differentiation, Ki-67 proliferation index, T stage, N stage, TNM stage, vascular invasion, and perineural invasion.

All cases were uniformly restaged according to the American Joint Committee on Cancer (AJCC) 8th edition TNM staging system. According to this system, TNM stage I was defined as T1-2, N0, M0. Given that only a small number of patients (*n* = 7) had received neoadjuvant therapy, pathological staging was based on postoperative histopathological examination in all cases. Preoperative serum levels of CA19-9 and CEA were measured within one week prior to surgery. For patients presenting with obstructive jaundice, serum CA19-9 levels were reassessed following biliary drainage to minimize the confounding effect of cholestasis on this biomarker.

### Follow up

Patients were regularly followed up after curative-intent resection. Overall survival (OS) was calculated from the date of surgery to the date of death or last follow-up, whichever occurred first. Recurrence-free survival (RFS) was defined as the interval from surgery to tumor recurrence or last follow-up. The final follow-up date was March 30, 2022.

### Propensity score matching (PSM)

Propensity scores were generated from a multivariable logistic regression model incorporating the following prespecified covariates: age, gender, preoperative serum CA19-9 and CEA levels, tumor location, surgical procedure, major postoperative complications, T stage, N stage, TNM stage, perineural invasion, lymphovascular invasion, and postoperative adjuvant chemotherapy. Matching was performed using a 1:2 nearest-neighbor protocol without replacement, with a caliper width set at 0.2 times the standard deviation of the logit-transformed propensity score.

### Statistical analysis

Patients were stratified into two groups according to the presence of predefined pathological risk factors. Tumors exhibiting poor differentiation or a Ki-67 index > 30% were assigned to the positive risk factor group (PRF), whereas those with well-to-moderate differentiation and a Ki-67 index ≤ 30% were assigned to the negative risk factor group (NRF). By comparing clinicopathological data between the two groups, we evaluated the impact of these pathological features on the efficacy of postoperative adjuvant chemotherapy, aiming to provide a scientific rationale for individualized treatment strategies.

Statistical analyses were performed using IBM SPSS Statistics (version 27.0; IBM Corp., Armonk, NY, USA), MedCalc (version 20), and R software (version 4.6.1). Continuous variables were expressed as median with interquartile range (IQR) and compared between groups using the Mann–Whitney *U*-test. Categorical variables were presented as frequencies with percentages and compared using the Chi-square test or Fisher's exact test, as appropriate. OS and RFS estimated using the Kaplan–Meier method, and survival curves were generated; between-group differences were evaluated with the log-rank test. Univariate and multivariate Cox proportional hazards regression analyses were performed to identify independent prognostic factors, with hazard ratios (HRs) and 95% confidence intervals (CIs) reported, and the Schoenfeld formula was employed to assess the statistical reliability of this finding. A two-tailed *p* < 0.05 was considered statistically significant.

## Results

### Clinicopathological features

The patient selection process is illustrated in Figure 1. After excluding 316 patients, a total of 520 patients were enrolled for analysis, among these, 32 (6.2% of the entire cohort) patients were diagnosed with pancreatic cancer through the community-based screening program. The variance inflation factor (VIF) for all covariates in the final multivariable model ranged from 1.12 to 1.48, indicating no significant multicollinearity (Supplementary Table S1). The overall events-per-variable (EPV) ratio was 31.55, exceeding the recommended minimum of 10.

**Figure 1:**
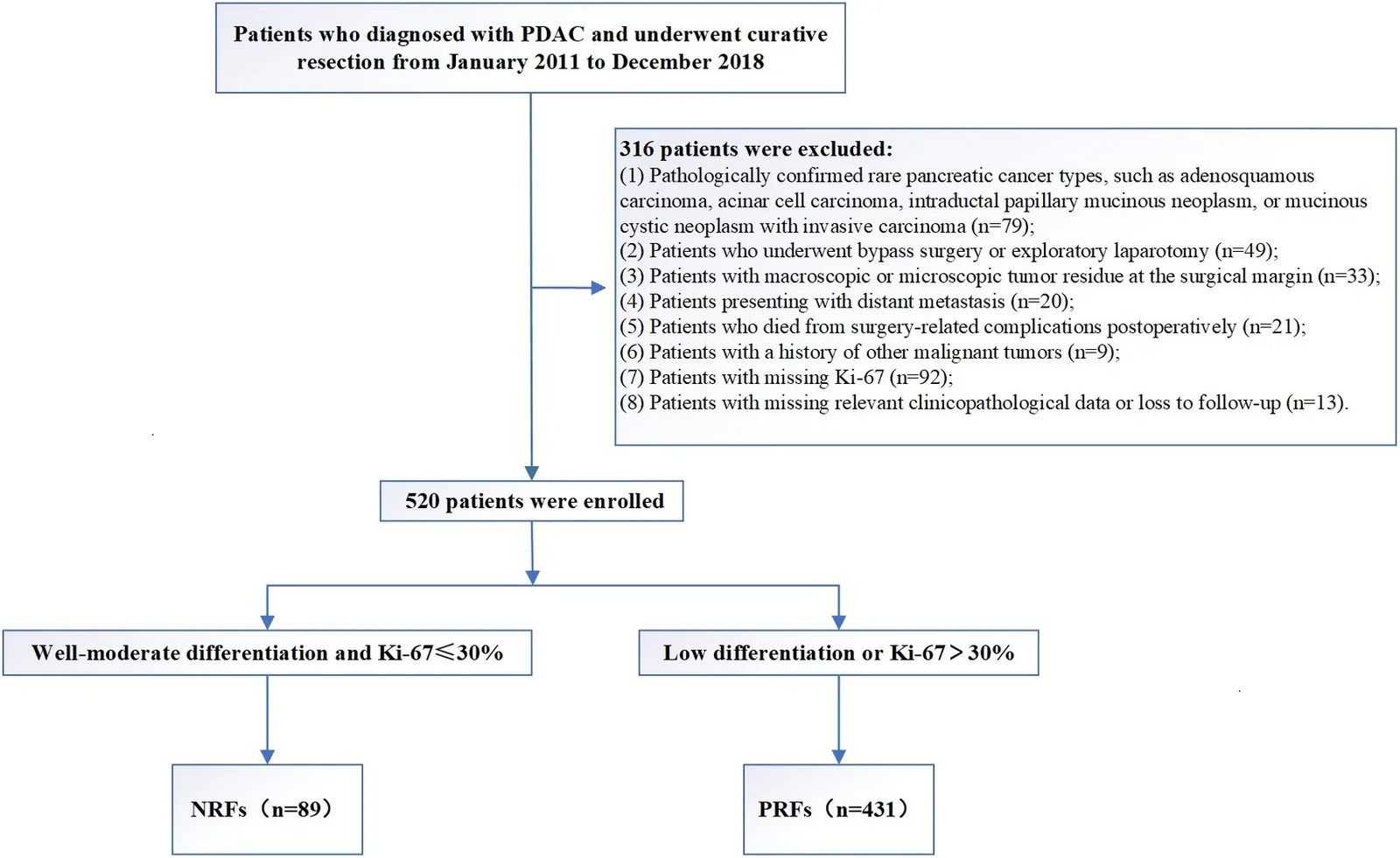
The inclusion and exclusion criteria of the study.

The baseline characteristics of the 520 enrolled patients are summarized as follows. The median age was 61 years (IQR: 55–67), and 225 patients (42.5%) were female. Surgical procedures included pancreaticoduodenectomy (PD) in 351 patients (67.5%) and distal pancreatectomy (DP) in 169 (32.5%). Notably, 21 patients (4.1%) underwent radical resection with portal vein or superior mesenteric vein resection and reconstruction. Postoperative complications occurred in 134 patients (25.8%), all of whom recovered with conservative management.

A total of 265 patients (51.0%) received adjuvant chemotherapy following surgery. The reasons for not receiving postoperative chemotherapy in the remaining patients are summarized in Supplementary Table S2. The chemotherapeutic regimens included FOLFIRINOX/mFOLFIRINOX, nab-paclitaxel plus gemcitabine (AG), nab-paclitaxel plus S-1 (AS), S-1 plus gemcitabine (GS), gemcitabine plus oxaliplatin (GEMOX), S-1 monotherapy, and gemcitabine monotherapy. The distribution of regimens was as follows: GS (*n* = 75), GEMOX (*n* = 54), gemcitabine (*n* = 45), S-1 (*n* = 45), AG (*n* = 33), FOLFIRINOX/mFOLFIRINOX (*n* = 12), and AS (*n* = 1). Patients received a median of 6 chemotherapy cycles (range: 3–12). In addition, we also performed an exploratory subgroup analysis stratified by specific chemotherapy regimens, and the results are presented in Supplementary Table S3.

According to the grouping criteria, the cohort was divided into the negative risk factor (NRF) group (*n* = 89, 17.1%) and the positive risk factor (PRF) group (*n* = 431, 82.9%). The clinicopathological characteristics of both groups, before and after propensity score matching, are summarized in Table 1, and the clinicopathological characteristics of patients with AJCC 8th TNM stage I are presented in Supplementary Table S4. Overall, the PRF group exhibited a significantly higher prevalence of vascular invasion (*x*^2^ = 15.856, *p* < 0.001) and more advanced T stage (*x*^2^ = 14.511, *p* = 0.002), N stage (*x*^2^ = 12.741, *p* = 0.002), and TNM stage (*x^2^* = 16.842, *p* = 0.002) compared with the NRF group. Conversely, no notable intergroup differences were observed for demographic variables (gender and age), serum tumor markers (CA19-9 and CEA), surgical parameters (tumor location, resection type, and postoperative complications), pathological feature (perineural infiltration), or administration of adjuvant chemotherapy (all *p* > 0.05). After propensity score matching, 86 patients in the NRF group were successfully matched to 170 patients in the PRF group at a 1:2 ratio. All covariates were well balanced between the two matched groups, with standardized mean differences below 0.1 (Table 1). The propensity score distribution before and after matching is depicted in Supplementary Figure S1.

**Table 1:** The clinicopathological characteristics of patients with PDAC according to the status of pathological risk factor.

Characteristic	Before matched		*p*	After matched		*p*
	Pathological risk factor			Pathological risk factor		
	No, *N* = 89, *n* (%)	Yes, *N* = 431, *n* (%)		No, *N* = 86, *n* (%)	Yes, *N* = 170, *n* (%)	
Gender			0.908			0.744
Male	50 (56.2)	245 (56.8)		48 (55.8)	100 (58.8)	
Female	39 (43.8)	186 (43.2)		38 (44.2)	70 (41.2)	
Age (years)			0.324			1.000
< 70	73 (82.0)	371 (86.1)		71 (82.6)	139 (81.8)	
≥ 70	16 (18.0)	60 (14.6)		15 (17.4)	31 (18.2)	
Preoperative CA19–9 (U/mL)			0.275			0.999
< 200	54 (60.7)	230 (53.4)		52 (60.5)	103 (60.6)	
200–1,000	26 (29.2)	131 (30.4)		25 (29.1)	49 (28.8)	
≥ 1,000	9 (10.1)	70 (16.2)		9 (10.5)	18 (10.6)	
Preoperative CEA (ng/mL)			0.188			0.619
≤ 5	67 (75.3)	294 (68.2)		64 (74.4)	120 (70.6)	
> 5	22 (24.7)	137 (31.8)		22 (25.6)	50 (29.4)	
Tumor location			0.541			0.995
Head	63 (70.8)	287 (66.6)		24 (27.9)	49 (28.8)	
Body and tail	26 (29.2)	144 (33.4)		62 (72.1)	121 (71.2)	
Type of pancreatectomy			0.487			1.000
PD	63 (70.8)	285 (66.1)		62 (72.1)	122 (71.8)	
DP	26 (29.2)	146 (33.9)		24 (27.9)	48 (28.2)	
T stage			0.002			0.661
T1	23 (25.8)	56 (13.0)		22 (25.6)	32 (18.8)	
T2	49 (55.1)	229 (53.1)		49 (57.0)	107 (62.9)	
T3	14 (15.7)	135 (31.3)		13 (15.1)	27 (15.9)	
T4	3 (3.4)	11 (2.6)		2 (2.3)	4 (2.4)	
N stage			0.002			0.998
N0	62 (69.7)	220 (51.0)		59 (68.6)	116 (68.2)	
N1	23 (25.8)	143 (33.2)		23 (26.7)	46 (27.1)	
N2	4 (4.5)	68 (15.8)		4 (4.7)	8 (4.7)	
TNM stage			0.001			0.938
I	49 (55.1)	142 (32.9)		48 (55.8)	91 (53.5)	
II	33 (37.1)	210 (48.7)		32 (37.2)	67 (39.4)	
III	7 (7.9)	79 (18.4)		6 (7.0)	12 (7.1)	
Perineural invasion			0.098			0.326
Absent	39 (43.8)	149 (34.6)		36 (41.9)	59 (34.7)	
Present	50 (56.2)	282 (65.4)		50 (58.1)	111 (65.3)	
Lymphovascular invasion			0.001			0.830
Absent	83 (93.3)	318 (73.8)		78 (90.7)	157 (92.4)	
Present	6 (6.7)	113 (26.2)		8 (9.3)	13 (7.6)	
Postoperative complications			0.189			0.641
Present	71 (20.2)	315 (73.1)		68 (79.1)	140 (82.4)	
Absent	18 (79.8)	116 (26.9)		18 (20.9)	30 (17.6)	
Postoperative adjuvant chemotherapy			0.189			1.000
No	38 (42.7)	217 (50.3)		48 (55.8)	94 (55.3)	
Yes	51 (57.3)	214 (49.7)		38 (44.2)	76 (44.7)	

Based on propensity score matching (1:2).

### Survival analysis of patients with PDAC

The results of univariate and multivariate analyses of factors influencing OS are summarized in Table 2. Multivariate Cox regression analysis identified age, preoperative serum CA19-9 level, pathological risk group, TNM stage, postoperative adjuvant chemotherapy, and postoperative complications as independent prognostic factors for OS. Notably, patients in the PRF group had significantly shorter OS compared with those in the NRF group (median OS: 19.0 months vs. 40.0 months; *p* < 0.001; Figure 2A). Stratified analysis of the overall cohort demonstrated that adjuvant chemotherapy provided a significant survival benefit in both the NRF group (HR = 0.577, 95% CI: 0.309–1.077, *p* = 0.021) and the PRF group (HR = 0.554, 95% CI: 0.442–0.694, *p* < 0.001) (Supplementary Table S5).

**Table 2:** Univariate and multivariate analyses of factors associated with OS and RFS in the entire study (*n* = 520).

Characteristics	*n*	OS				RFS			
		Univariate analysis		Multivariate analysis		Univariate analysis		Multivariate analysis	
		HR (95%CI)	*p*	HR (95%CI)	*p*	HR (95%CI)	*p*	HR (95%CI)	*p*
Gender									
Male	295	1 (ref)				1 (ref)			
Female	225	0.912 (0.736–1.130)	0.400			0.931 (0.763–1.136)	0.483		
Age (years)									
<70	444	1 (ref)		1 (ref)		1 (ref)			
≥ 70	76	1.421 (1.067–1.892)	0.016	1.420 (1.059–1.905)	0.019	1.142 (0.864–1.509)	0.352		
Tumor location									
Head	350	1 (ref)				1 (ref)			
Body and tail	170	1.021 (0.813–1.282)	0.859			0.993 (0.804–1.227)	0.951		
Preoperative CEA (ng/mL)									
≤ 5.0	361	1 (ref)		1 (ref)		1 (ref)		1 (ref)	
> 5.0	159	1.386 (1.105–1.738)	0.005	1.166 (0.907–1.498)	0.232	1.382 (1.118–1.708)	0.003	1.207 (0.956–1.525)	0.113
Preoperative CA19–9 (U/mL)									
<200.0	284	1 (ref)		1 (ref)		1 (ref)		1 (ref)	
200.0–1,000.0	157	1.322 (1.041–1.680)	0.022	1.385 (1.081–1.776)	0.010	1.363 (1.090–1.703)	0.007	1.323 (1.051–1.666)	0.017
≥ 1,000.0	79	1.875 (1.398–2.514)	< 0.001	1.477 (1.083–2.015)	0.014	2.182 (1.658–2.871)	< 0.001	1.753 (1.310–2.345)	＜0.001
TNM stage									
I	191	1 (ref)		1 (ref)		1 (ref)		1 (ref)	
II	243	1.618 (1.271–2.061)	< 0.001	1.391 (1.051–1.841)	0.021	1.489 (1.191–1.862)	0.004	1.379 (1.042–1.826)	0.025
III	86	2.449 (1.806–3.322)	< 0.001	2.285 (1.698–3.075)	< 0.001	2.519 (1.775–3.142)	< 0.001	2.286 (1.698–3.077)	< 0.001
Lymphovascular invasion									
Absent	381	1 (ref)		1 (ref)		1 (ref)		1 (ref)	
Present	139	1.383 (1.097–1.743)	0.006	0.819 (0.627–1.069)	0.142	1.596 (1.289–1.977)	< 0.001	0.971 (0.759–1.243)	0.818
Perineural invasion									
Absent	183	1 (ref)		1 (ref)		1 (ref)		1 (ref)	
Present	337	1.377 (1.098–1.727)	0.006	1.094 (0.865–1.383)	0.261	1.404 (1.136–1.736)	0.002	1.190 (0.957–1.497)	0.117
Pathological risk factors									
Absent	89	1 (ref)		1 (ref)		1 (ref)		1 (ref)	
Present	431	2.338 (1.680–3.253)	< 0.001	1.906 (1.355–2.680)	< 0.001	2.338 (1.680–3.253)	＜0.001	1.702 (1.258–2.303)	＜0.001
Postoperative complications									
Absent	386	1 (ref)		1 (ref)		1 (ref)		1 (ref)	
Present	134	1.539 (1.215–1.950)	< 0.001	1.386 (1.083–1.773)	0.010	1.380 (1.105–1.723)	0.004	1.176 (0.930–1.486)	0.176
Postoperative adjuvant chemotherapy									
No	255	1 (ref)		1 (ref)		1 (ref)		1 (ref)	
Yes	265	0.552 (0.446–0.628)	< 0.001	0.518 (0.417–0.645)	< 0.001	0.732 (0.601–0.890)	0.002	0.736 (0.602–0.900)	0.003

**Figure 2:**
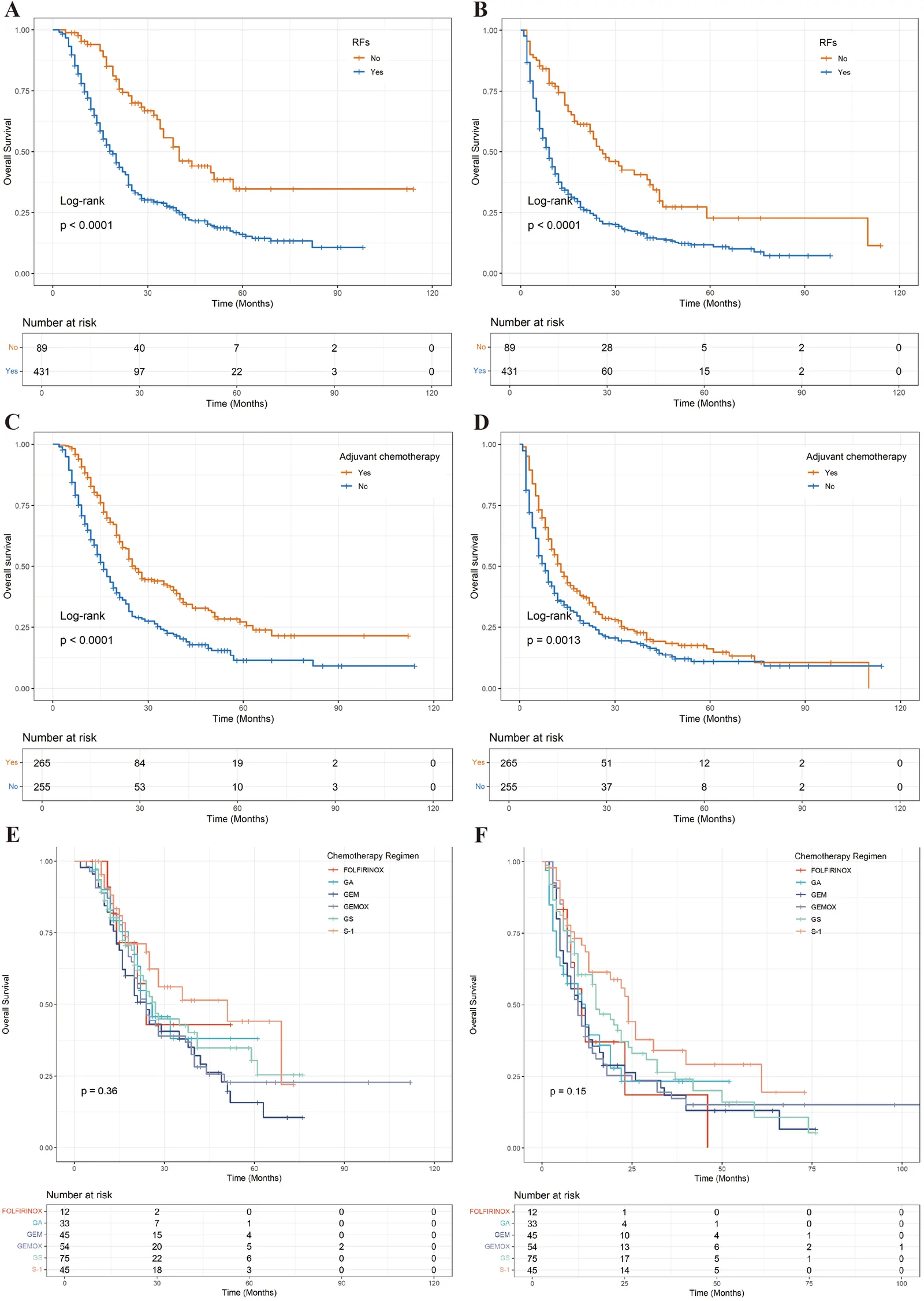
Patients in the PRF group had shorter OS **(A)** and RFS **(B)** compared with surgery alone, adjuvant chemotherapy was associated with a significant improvement in OS **(C)** and RFS **(D)** there was no significant difference on the choice of chemotherapy regimen in OS **(E)** and RFS **(F)**.

Similarly, multivariate analysis identified higher preoperative CA19-9 level, presence of pathological risk factors, advanced TNM stage, and lack of adjuvant chemotherapy as independent unfavorable prognostic factors for RFS (Table 2). Patients in the PRF group had significantly shorter RFS than those in the NRF group (median RFS: 9.0 months vs. 26.0 months; *P* < 0.001; Figure 2B).

Compared with surgery alone, adjuvant chemotherapy was associated with significantly improved OS (median OS: 26.0 months vs. 16.0 months; *p* < 0.001; Figure 2C) and RFS (median RFS: 13.0 months vs. 8.0 months; *p* = 0.001; Figure 2D). However, no significant difference in OS (*p* = 0.425; Figure 2E) or RFS (*p* = 0.164; Figure 2F) was observed among patients receiving different chemotherapy regimens.

In addition, in the TNM stage I/NRF subgroup (*n* = 49), 28 patients (57.1%) received adjuvant chemotherapy and 21 (42.9%) did not. Adjuvant chemotherapy was not associated with a significantly improved overall survival compared with no chemotherapy, with an adjusted HR of 0.491 (95% CI: 0.186–1.298, *p* = 0.365). We performed a *post-hoc* power calculation using the Schoenfeld formula, the calculated power was approximately 31.1%.

### The impact of adjuvant chemotherapy on patients in PRFs

The results of univariate and multivariate Cox regression analyses of factors affecting OS and RFS in the PRF group are summarized in Table 3. Multivariate analysis identified advanced age, elevated preoperative CA19-9, advanced TNM stage, presence of postoperative complications, and lack of adjuvant chemotherapy as independent risk factors for OS. Similarly, elevated CA19-9, advanced TNM stage, postoperative complications, and omission of adjuvant chemotherapy were independently associated with worse RFS.

**Table 3:** Univariate and multivariate analysis of factors associated with OS and RFS in PRFs.

Characteristics	OS				RFS			
	Univariate cox analysis		Multivariate cox analysis		Univariate cox analysis		Multivariate cox analysis	
	HR (95% CI)	*p*	HR (95% CI)	*p*	HR (95% CI)	*p*	HR (95% CI)	*p*
Gender								
Male	1 (ref)				1 (ref)			
Female	0.897 (0.714–1.1,228)	0.352			0.936 (0.756–1.159)	0.544		
Age (years)								
< 70	1 (ref)		1 (ref)		1 (ref)			
≥ 70	1.478 (1.082–2.018)	0.014	1.581 (1.150–2.175)	0.005	1.150 (0.847–1.560)	0.370		
Preoperative serum CA19–9 (U/mL)								
< 200.0	1 (ref)		1 (ref)		1 (ref)		1 (ref)	
200.0–1,000.0	1.291 (1.000–1.666)	0.050	1.417 (1.087–1.846)	0.010	1.354 (1.064–1.722)	0.014	1.363 (1.065–1.746)	0.014
≥ 1,000.0	1.706 (1.252–2.323)	＜0.001	1.507 (1.088–2.088)	0.014	2.041 (1.526–2.730)	＜0.001	1.802 (1.325–2.452)	＜0.001
Preoperative serum CEA (ng/mL)								
≤ 5	1 (ref)		1 (ref)		1 (ref)		1 (ref)	
> 5	1.290 (1.014–1.641)	0.038	1.520 (0.718–3.220)	0.274	1.269 (1.012–1.592)	0.039	1.117 (0.877–1.424)	0.370
Tumor location								
Head	1 (ref)				1 (ref)			
Body and tail	1.005 (0.789–1.280)	0.969			1.028 (0.821–1.288)	0.810		
Operation type								
PD	1 (ref)				1 (ref)			
DP	1.044 (0.820–1.327)	0.728			1.070 (0.855–1.339)	0.554		
TNM stage								
I	1 (ref)		1 (ref)		1 (ref)		1 (ref)	
II	1.431 (1.105–1.855)	0.007	1.293 (0.989–1.691)	0.060	1.418 (1.110–1.810)	0.005	1.245 (0.969–1.599)	0.086
III	2.077 (1.503–2.870)	＜0.001	2.120 (1.519–2.959)	＜0.001	2.182 (1.606–2.964)	＜0.001	2.047 (1.504–2.787)	＜0.001
Perineural invasion								
Absence	1 (ref)				1 (ref)			
Presence	1.153 (0.909–1.463)	0.241			1.196 (0.955–1.498)	0.120		
Lymphovascular invasion								
Absence	1 (ref)				1 (ref)			
Presence	1.052 (0.817–1.355)	0.695			1.249 (0.989–1.578)	0.062		
Postoperative adjuvant chemotherapy								
No	1 (ref)		1 (ref)		1 (ref)		1 (ref)	
Yes	0.554 (0.441–0.694)	＜0.001	0.520 (0.4,131–0.656)	＜0.001	0.699 (0.566–0.863)	＜0.001	0.685 (0.553–0.850)	＜0.001
Postoperative complications								
Absence	1 (ref)		1 (ref)		1 (ref)		1 (ref)	
Presence	1.584 (1.236–2.029）	＜0.001	1.555 (1.206–2.005)	＜0.001	1.384 (1.094–1.751)	0.007	1.294 (1.016–1.648)	0.037

In addition, we further evaluated the impact of adjuvant chemotherapy on survival across different TNM stages within the PRF group. In the overall PRF cohort, patients in PRF group could confer a survival benefit from postoperative adjuvant chemotherapy (5-year OS: 9.3% vs. 23.0%; 5-year RFS: 8.5% vs. 14.9%). However, the magnitude of this benefit varied significantly by TNM stage (Figure 3). For patients with AJCC 8th TNM stage I, adjuvant chemotherapy significantly improved both OS (median OS: 28.0 months vs. 17.0 months, *p* < 0.001) (Figure 3A) and RFS (median RFS: 21.0 months vs. 7.0 months, *p* = 0.004) (Figure 3D). In contrast, for patients with AJCC 8th TNM stage II or TNM stage III, adjuvant chemotherapy was associated with improved OS [TNM stage II median OS: 22.0 months vs. 14.0 months, *p* = 0.002 (Figure 3B); TNM stage III median OS: 18.0 months vs. 10.0 months, *p* = 0.004 (Figure 3C)] but did not significantly prolong RFS [TNM stage II median RFS: 10.0 months vs. 7.0 months, *p* = 0.991 (Figure 3E); TNM stage III median RFS: 6.0 months vs. 3.0 months, *p* = 0.209 (Figure 3F)].

**Figure 3:**
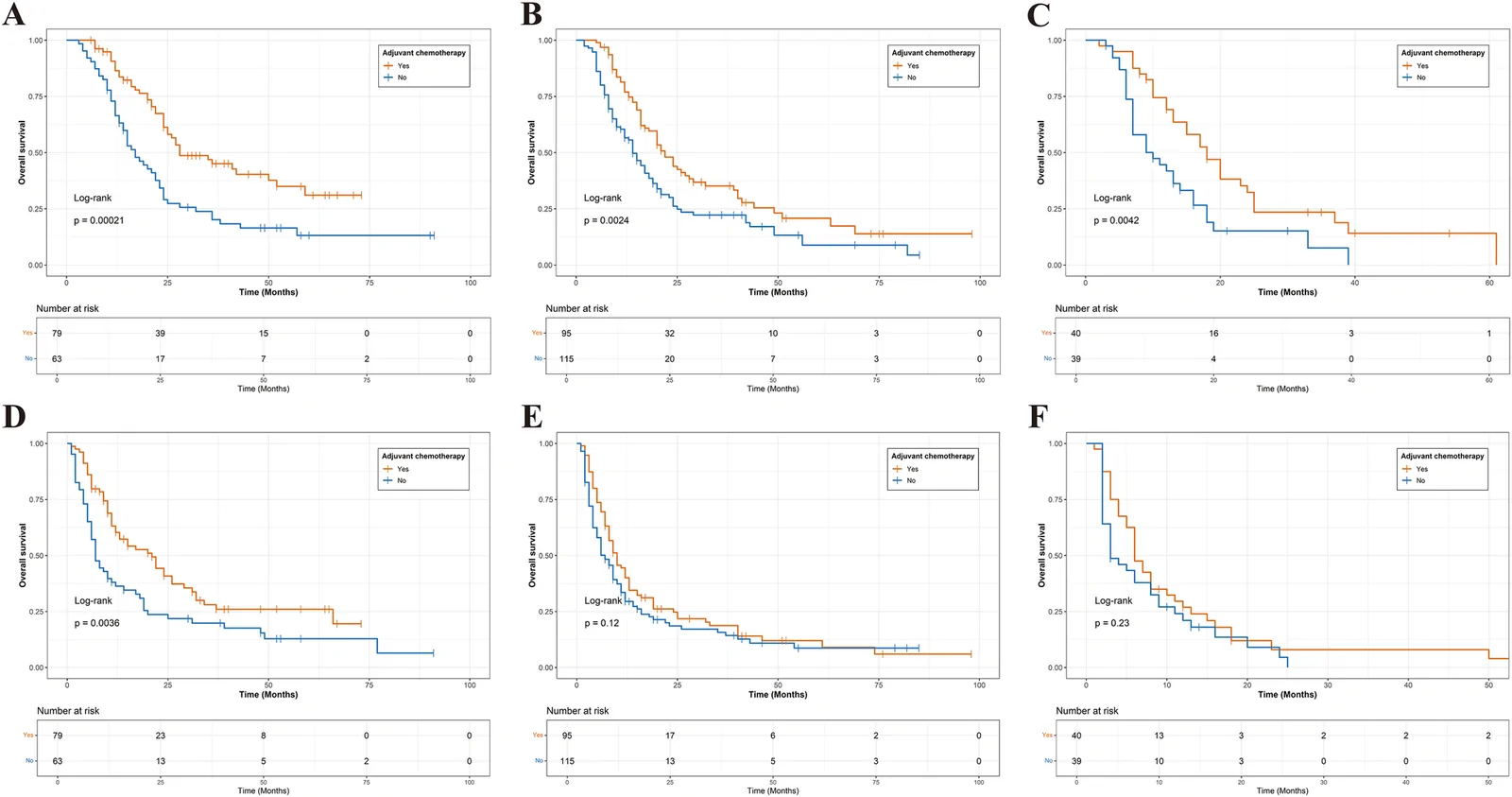
Impact of postoperative adjuvant chemotherapy on OS and RFS in patients with different TNM stage in PRFs. **(A)** Stage Ⅰ OS; **(B)** Stage Ⅱ OS; **(C)** Stage Ⅲ OS; **(D)** Stage Ⅰ RFS; **(E)** Stage Ⅱ RFS; **(F)** Stage Ⅲ RFS.

### The impact of adjuvant chemotherapy on patients in NRFs

The results of univariate and multivariate analyses of factors affecting OS and RFS in patients without pathological risk factors (NRF group) are summarized in Table 4. In univariate analysis, preoperative CA19-9 ≥1,000 U/mL, CEA > 5 ng/mL, advanced TNM stage, and perineural invasion were identified as unfavorable factors for OS; however, none of these variables retained statistical significance in the multivariate model. For RFS, univariate analysis identified CA19-9 ≥ 1,000 U/mL, CEA > 5 ng/mL, perineural invasion, and lymphovascular invasion as adverse prognostic factors. Among these, CA19-9 ≥ 1,000 U/mL (HR = 2.926, 95% CI: 1.131–7.567; *p* = 0.039) and perineural invasion (HR = 2.068, 95% CI: 1.106–3.865; *p* = 0.023) remained independent risk factors for RFS in the multivariate analysis.

**Table 4:** Univariate and multivariate analysis of factors associated with OS and RFS in NRFs.

Characteristics	OS				RFS			
	Univariate cox analysis		Multivariate cox analysis		Univariate cox analysis		Multivariate cox analysis	
	HR (95% CI)	*p*	HR (95% CI)	*p*	HR (95% CI)	*p*	HR (95% CI)	*p*
Gender								
Male	1 (ref)				1 (ref)			
Female	0.670 (0.515–1.815)	0.917			1.170 (0.670–2.043)	0.582		
Age (years)								
< 70	1 (ref)				1 (ref)			
≥ 70	2.162 (0.998–4.684)	0.051			1.484 (0.738–2.948)	0.268		
Preoperative serum CA19–9 (U/mL)								
< 200.0	1 (ref)		1 (ref)		1 (ref)		1 (ref)	
200.0–1,000.0	1.568 (0.791–3.110)	0.198	1.289 (0.622–2.672)	0.495	1.443 (0.795–2.617)	0.228	1.380 (0.738–2.580)	0.314
≥ 1,000.0	3.036 (1.185–7.781)	0.021	2.332 (0.840–6.479)	0.104	3.112 (1.324–7.314)	0.009	2.926 (1.131–7.567)	0.039
Preoperative serum CEA (ng/mL)								
≤ 5	1 (ref)		1 (ref)		1 (ref)		1 (ref)	
> 5	2.041 (1.043–3.993)	0.037	1.520 (0.718–3.220)	0.274	2.104 (1.151–3.846)	0.016	1.445 (0.727–2.873)	0.294
Tumor location								
Head	1 (ref)				1 (ref)			
Body and tail	1.448 (0.714–2.934)	0.238			0.805 (0.435–1.491)	0.490		
Operation type								
PD	1 (ref)				1 (ref)			
DP	1.105 (0.558–2.186)	0.482			0.870 (0.475–1.591)	0.650		
TNM stage								
I	1 (ref)		1 (ref)		1 (ref)			
II	2.121 (1.079–4.169)	0.029	1.864 (0.922–3.768)	0.083	1.278 (0.709–2.306)	0.414		
III	3.300 (1.285–8.472)	0.013	2.132 (0.751–6.005)	0.156	2.113 (0.861–5.185)	0.102		
Perineural invasion								
Absence	1 (ref)		1 (ref)		1 (ref)		1 (ref)	
Presence	2.373 (1.214–4.641)	0.012	1.798 (0.846–3.817)	0.127	2.240 (1.240–4.046)	0.002	2.068 (1.106–3.865)	0.023
Lymphovascular invasion								
Absence	1 (ref)				1 (ref)		1 (ref)	
Presence	1.915 (0.749–4.899)	0.175			2.708 (1.143–6.411)	0.024	2.154 (0.863–5.374)	0.100
Postoperative adjuvant chemotherapy								
No	1 (ref)				1 (ref)			
Yes	0.577 (0.309–1.078)	0.084			1.128 (0.650–1.956)	0.669		
Postoperative complications								
Absence	1 (ref)				1 (ref)			
Presence	0.867 (0.363–2.072)	0.748			1.049 (0.525–2.098)	0.892		

Furthermore, the impact of adjuvant chemotherapy on survival in the NRF group varied according to TNM stage (Figure 4). For patients with AJCC 8th TNM stage I, adjuvant chemotherapy did not significantly improve either OS (median: not reached vs. 50.0 months; *p* = 0.365; Figure 4A) or RFS (median: 24.0 vs. 44.0 months; *p* = 0.569; Figure 4D). In contrast, for patients with AJCC 8th TNM stage II or TNM stage III disease, adjuvant chemotherapy significantly prolonged OS [stage II: 51.0 vs. 32.0 months, *p* = 0.041 (Figure 4B); stage III: 38.0 vs. 25.0 months, *p* = 0.047 (Figure 4C)], but failed to significantly improve RFS [stage II: 22.0 vs. 25.0 months, *p* = 0.991, (Figure 4E); stage III: 17.0 vs. 18.0 months, *p* = 0.446 (Figure 4F)]. After matching, in the AJCC 8th TNM stage I/NRF subgroup, adjuvant chemotherapy remained not significantly associated with OS (HR = 1.695, 95% CI 0.664–4.323, *p* = 0.270) or RFS (HR = 1.204, 95% CI 0.367–1.734, *p* = 0.644) in the propensity-matched analysis (Supplementary Table S6).

**Figure 4:**
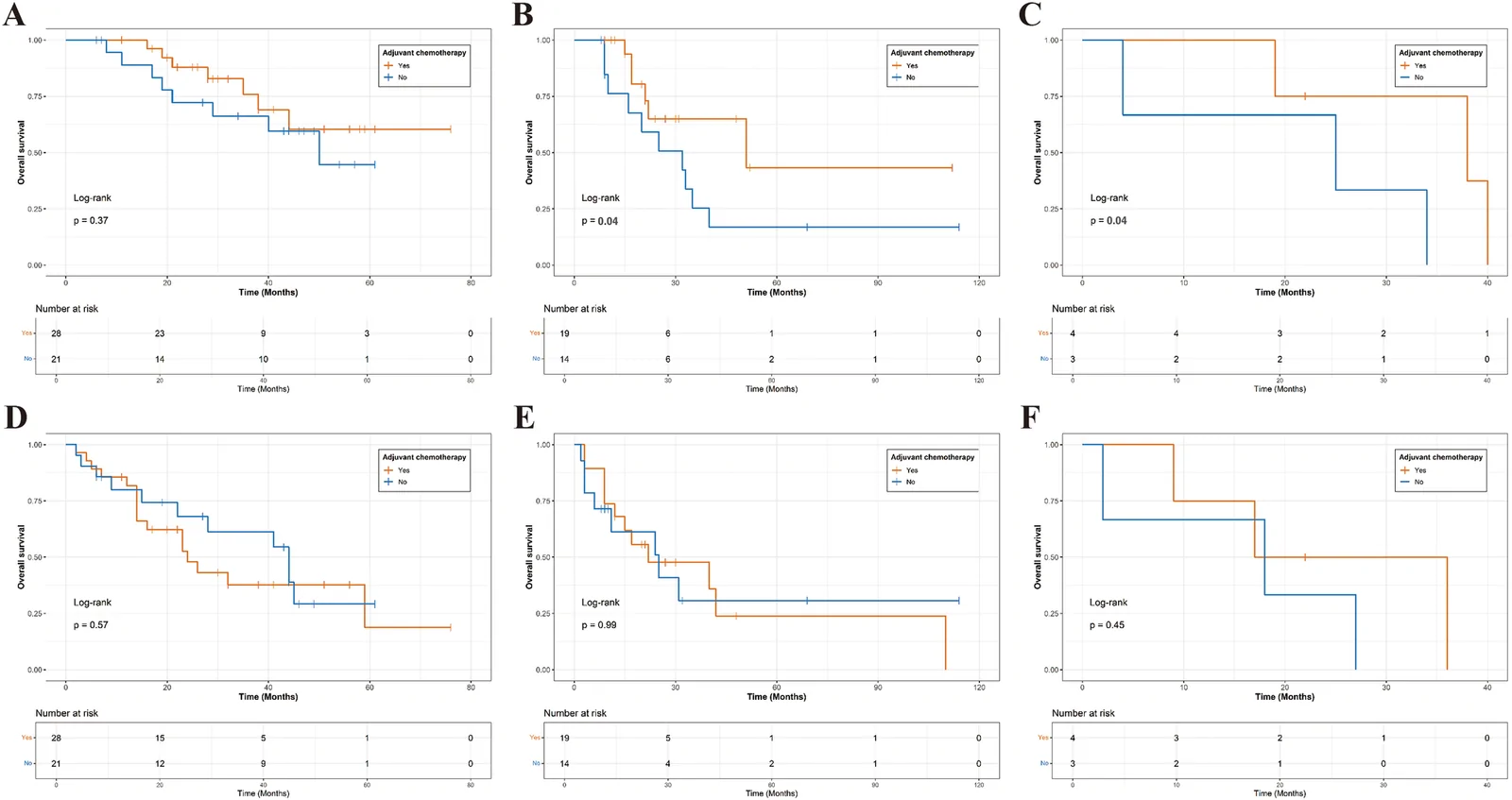
Impact of postoperative adjuvant chemotherapy on OS and RFS in patients with different TNM stage in NRFs. **(A)** Stage Ⅰ OS; **(B)** Stage Ⅱ OS; **(C)** Stage Ⅲ OS; **(D)** Stage Ⅰ RFS; **(E)** Stage Ⅱ RFS; **(F)** Stage Ⅲ RFS.

## Discussion

The prognostic value and clinical applicability of the eighth edition AJCC TNM staging system for pancreatic cancer have been validated by several large-scale studies (16–18). Multiple prospective trials have confirmed that adjuvant chemotherapy significantly prolongs survival in patients with pancreatic cancer (3–7), and several clinical practice guidelines recommend systemic adjuvant chemotherapy for PDAC patients regardless of TNM stage (17, 19, 20). However, the efficacy of postoperative adjuvant chemotherapy is influenced by various factors, including patients' physical status, tolerance, and sensitivity to the specific regimen (21). Currently, gemcitabine-based regimens remain the backbone of first-line adjuvant therapy, including gemcitabine monotherapy, gemcitabine plus capecitabine, and gemcitabine plus nab-paclitaxel (4, 7). In addition, the FOLFIRINOX regimen has gradually become a mainstream option owing to its demonstrated efficacy (22).

Our findings suggest that not all patients with PDAC necessarily benefit from postoperative adjuvant chemotherapy. Although adjuvant chemotherapy significantly improved both OS and RFS in the overall cohort (both *p* < 0.001), stratified analysis by TNM stage and pathological risk status revealed considerable heterogeneity in treatment response. Specifically, for patients with AJCC 8th TNM stage I disease lacking pathological risk factors (NRF group), adjuvant chemotherapy did not significantly prolong either OS (median: not reached vs. 50.0 months; *p* = 0.365) or RFS (median: 24.0 vs. 44.0 months; *p* = 0.569). In contrast, for TNM stage I patients with pathological risk factors (PRF group), adjuvant chemotherapy conferred significant benefits in both OS (median: 28.0 vs. 17.0 months; *p* < 0.001) and RFS (median: 21.0 vs. 7.0 months; *p* = 0.004). For patients with TNM stage II or III disease, adjuvant chemotherapy significantly improved OS regardless of pathological risk status, but its benefit on RFS was limited. Collectively, these results indicate that the survival benefit of adjuvant chemotherapy is closely associated with both TNM stage and pathological background. Although current clinical guidelines emphasize the importance of systemic adjuvant chemotherapy, whether routine administration is warranted for patients with stage I disease remains controversial.

Multiple studies have confirmed that adjuvant chemotherapy after surgery significantly improves prognosis in patients with stage I PDAC compared with surgery alone (1, 9, 10, 23). However, a meta-analysis suggested that the benefit of postoperative adjuvant chemotherapy in this population may be limited to short-term survival (8). Of note, these studies did not fully account for the biological heterogeneity of PDAC. In a stratified analysis based on pathological features, Zou et al. found that adjuvant chemotherapy did not improve prognosis in patients without perineural or lymphovascular invasion (24). Similarly, Ryoga Hamura et al. demonstrated that adjuvant chemotherapy conferred no survival benefit in stage I patients without extrapancreatic invasion (25). More recently, Izumo et al. stratified stage IA patients into low- and high-risk groups based on clinicopathological factors and observed that the high-risk group derived significantly greater benefit from adjuvant chemotherapy (5-year RFS: 32% with chemotherapy vs. 0% without; *p* = 0.045), whereas the benefit was minimal in the low-risk group (5-year RFS: 75% vs. 70%; *p* = 0.49) (26).

In the present study, we performed a stratified analysis incorporating pathological risk factors, including Ki-67 expression and tumor differentiation, in patients with PDAC. Among TNM stage I patients with pathological risk factors, adjuvant chemotherapy significantly improved both OS (median: 28.0 vs. 17.0 months; *p* < 0.001) and RFS (median: 21.0 vs. 7.0 months; *p* = 0.004). By contrast, in TNM stage I patients without such risk factors, adjuvant chemotherapy conferred no significant survival benefit over surgery alone (median OS: not reached vs. 50.0 months, *p* = 0.365; median RFS: 24.0 vs. 44.0 months, *p* = 0.569). These findings align with the notion that tumor biological characteristics—rather than TNM stage alone—should guide adjuvant treatment decisions. Indeed, Gervaso et al. have emphasized that the decision regarding adjuvant therapy for stage I PDAC remains challenging and requires further evidence to reach definitive conclusions (27). Collectively, our results suggest that postoperative adjuvant chemotherapy should not be generalized to all TNM stage I patients; rather, its benefit is largely dependent on tumor biological features such as poor differentiation and perineural invasion. The presence or absence of pathological risk factors should therefore serve as a critical determinant in the selection of candidates for adjuvant chemotherapy. It was worth noticing that the *post-hoc* power for this comparison was only 31.1%, indicating that the study was insufficiently powered to detect the observed effect size. This is likely attributable to the limited number of events (*n* = 17) in this specific subgroup.

In contrast to the findings in TNM stage I disease, adjuvant chemotherapy significantly prolonged OS in patients with TNM stage II or III disease, irrespective of pathological risk status. Specifically, among NRF group, median OS was 51.0 vs. 32.0 months for stage II (*p* = 0.041) and 38.0 vs. 25.0 months for stage III (*p* = 0.047). Among PRF group, median OS was 32.0 vs. 22.0 months for stage II (*p* = 0.003) and 18.0 vs. 10.0 months for stage III (*p* = 0.004). Notably, the survival benefit of adjuvant chemotherapy appeared more pronounced in patients with pathological risk factors than in those without. This observation is consistent with previous reports in the literature. Toshitaka Sugawara et al. (28) and Abdulrahman Y. Hammad et al. (29) confirmed a significant correlation between adjuvant chemotherapy and improved survival, noting that even node-negative patients could derive benefit. In contrast, Roessel et al. (30) found that postoperative adjuvant chemotherapy improved survival only in patients with pathologically confirmed lymph node metastasis. Adding to this controversy, another study suggested that TNM stage II and node-negative patients benefited from adjuvant chemotherapy, whereas TNM stage III and node-positive patients did not (1), which was consistent with the results of the ESPAC4 phase III clinical trial (31). Furthermore, Zhang et al. reported that in patients with borderline resectable or locally advanced pancreatic cancer who received neoadjuvant therapy followed by surgery, postoperative adjuvant chemotherapy not only failed to improve survival after R0 resection but was also associated with shortened survival in node-positive patients (32). Incorporating pathological risk factors, Hammad et al. further demonstrated that adjuvant chemotherapy significantly prolonged survival in patients with perineural invasion but without lymph node metastasis, suggesting that perineural invasion may warrant adjuvant treatment (29). Similarly, other studies have indicated that the benefit of adjuvant chemotherapy is closely linked to tumor differentiation grade (33). Collectively, these findings underscore the complex and heterogeneous role of pathological risk factors in determining adjuvant chemotherapy benefit. Even among patients at the same TNM stage, the presence of different pathological features may lead to markedly divergent survival outcomes. This heterogeneity reflects the intrinsic biological complexity of PDAC and highlights the urgent need for further mechanistic studies to clarify how pathological risk factors modulate chemotherapy response, ultimately facilitating more precise individualized treatment strategies.

Although it is widely acknowledged that adjuvant chemotherapy significantly improves overall survival, its ability to delay early recurrence remains controversial. Li et al. (1) and Wu et al. (33) analyzed the survival benefits of adjuvant chemotherapy across different TNM stages and found that it did not improve RFS in patients with stage II or III disease. In contrast, other studies have reached conflicting conclusions. For instance, the multicenter, randomized controlled phase III CONKO-001 trial, which enrolled 368 patients, demonstrated that gemcitabine-based adjuvant chemotherapy significantly prolonged median RFS compared with surgery alone (34). Notably, none of the aforementioned studies systematically accounted for the potential impact of pathological risk factors on RFS outcomes.

In our study, which incorporated pathological risk factors into the analysis, we found that adjuvant chemotherapy did not significantly improve RFS in patients with TNM stage II or III PDAC, regardless of pathological risk status. Among NRF patients, median RFS was 22.0 vs. 25.0 months for stage II (*p* = 0.991) and 17.0 vs. 18.0 months for stage III (*p* = 0.446). Among PRF patients, median RFS was 10.0 vs. 7.0 months for stage II (*p* = 0.991) and 6.0 vs. 3.0 months for stage III (*p* = 0.209). These findings align with previous reports suggesting that the RFS benefit of adjuvant chemotherapy may be closely linked to pathological characteristics. For instance, one study demonstrated that in N0 patients with perineural invasion, adjuvant chemotherapy significantly prolonged RFS, whereas no such benefit was observed in those without perineural invasion (29). Amirian et al. (35) further confirmed that adjuvant chemotherapy could delay local recurrence and distant metastasis in lymph node-positive patients, irrespective of the number of positive nodes. Additionally, Kurahara et al. (36) found that adjuvant chemotherapy significantly prolonged RFS only in patients whose CA19-9 levels failed to normalize after neoadjuvant therapy, while no benefit was observed in those with lymph node metastasis. Collectively, these findings suggest that tailoring adjuvant chemotherapy regimens based on individual pathological profiles represents an important avenue for future investigation. Further studies are warranted to clarify the role of adjuvant chemotherapy across different pathological subgroups and to provide more precise guidance for clinical decision-making.

In our study, a total of 255 patients did not receive adjuvant chemotherapy, The primary reasons were patient preference/refusal (*n* = 57), and poor postoperative recovery/complications (*n* = 44). The third reason was poor performance status/comorbidities (*n* = 31). Only a small fraction (*n* = 14) was omitted due to physician's judgment of low-risk stage. Despite our best efforts to retrieve relevant information through chart review, sufficient clinical details were lacking in the records of some patients (*n* = 109), potential contributing factors included financial constraints, loss to follow-up, or transfer to other hospitals for subsequent management. Moreover, given the substantial time span over which the study cohort was enrolled, it is important to note that adjuvant chemotherapy was not established as a standard of care during the early phase of the study period, as its survival benefit had not yet been robustly validated by then-prevailing evidence-based guidelines. In addition, our study identified advanced age (HR = 1.672, 95% CI:1.243–2.249, *p* < 0.001) and postoperative complications (HR = 1.446, 95% CI:1.215–1.858, *p* = 0.004) as independent risk factors for OS. This may be partly attributable to the fact that patients with these factors were less likely to receive scheduled adjuvant chemotherapy. Previous studies have shown that advanced age is an important factor influencing the decision to administer postoperative adjuvant chemotherapy (37–39), potentially due to prolonged postoperative recovery, reduced tolerance to chemotherapy-related adverse effects, and a lower likelihood of clinical trial enrollment among older patients. Similarly, the occurrence of postoperative complications has been identified as a key determinant affecting chemotherapy decisions (40, 41). A French study reported that 40% of patients with PDAC did not receive adjuvant chemotherapy after surgery, and nearly 80% of those who underwent resection were unable to complete the planned regimen (38), with postoperative complications being the primary reason for omission or interruption. Beyond their direct impact on treatment delivery, postoperative complications may also induce systemic inflammatory responses and inflammation-associated immunosuppression, thereby contributing to a worse prognosis (42). Collectively, these findings underscore the urgent need for effective strategies to prevent and manage postoperative complications, thereby minimizing their negative impact on adjuvant chemotherapy delivery and improving long-term outcomes in this patient population.

Several limitations of this study should be acknowledged. First, as a single-center retrospective study with a relatively limited sample size, selection bias may have been introduced, potentially affecting the generalizability of our findings. Second, some patients did not receive or discontinued adjuvant chemotherapy for various reasons, which may have influenced the accuracy of our results. Although we pooled all adjuvant regimens for the primary analysis, we acknowledge that this approach may obscure regimen-specific differences in efficacy and tolerability; our exploratory subgroup analyses according to regimen type were underpowered due to small sample sizes and should therefore be interpreted with caution. Third, owing to the extremely small number of patients who received neoadjuvant therapy, we did not include this variable as a covariate in our statistical models, which may have partially confounded the results. Fourth, we did not collect data on post-recurrence treatment, which may have substantially affected overall survival. Consequently, external validation using data from diverse populations and regions is warranted. Multi-center and prospective studies are needed to confirm the robustness and generalizability of our findings. Future research should also incorporate additional variables, including post-recurrence treatment strategies, to more comprehensively evaluate the impact of adjuvant chemotherapy on patient prognosis.

## Conclusions

The present study demonstrates that pathological risk factors may play a key role in determining the benefit of postoperative adjuvant chemotherapy in patients with AJCC 8th TNM stage I PDAC. In our cohort, stage I patients with pathological risk factors (PRF group) derived significant improvements in both OS and RFS from adjuvant chemotherapy, whereas limited benefit was observed in those without these factors (NRF group).

Taken together, these findings suggest that the majority of patients with PDAC derive survival benefit from postoperative adjuvant chemotherapy. However, patients with TNM stage I disease who lack pathological risk factors did not show a clear advantage in this analysis. Therefore, for this specific subgroup, the decision to administer adjuvant chemotherapy should be made with careful consideration, as the balance between potential risks and the lack of a clearly established survival benefit remains uncertain. In addition, while our data suggest limited benefit of adjuvant chemotherapy in this population, the findings should be interpreted as exploratory, and larger prospective studies are warranted to confirm these observations.

## Data Availability

The original contributions presented in the study are included in the article/Supplementary Material, further inquiries can be directed to the corresponding authors.
