# Supplementary material for: TNM stage I pancreatic ductal adenocarcinoma patients without pathological risk may drive limited benefit from adjuvant chemotherapy
Source: Front Med (Lausanne). 2026 Sep 14;13:1914610. doi: 10.3389/fmed.2026.1914610 (PMC13618324; doi:10.3389/fmed.2026.1914610)
Supplement: Supplementary file 1 [file Table1.docx]

| Table S1. Multicollinearity assessment (VIF) and events-per-variable (EPV) ratio for the primary multivariable model | |
| --- | --- |
| **Covariate** | **VIF** |
| Gender | 1.008 |
| Age (years) | 1.032 |
| Tumor location | 1.036 |
| preoperative CEA (ng/mL) | 1.014 |
| preoperative CA19-9 (U/mL) | 1.033 |
| TNM stage | 1.265 |
| Lymphovascular invasion | 1.266 |
| Perineural invasion | 1.017 |
| Pathological risk factors | 1.053 |
| Postoperative complications | 1.037 |
| Postoperative adjuvant chemotherapy | 1.016 |
| **Overall model** | |
| Total events (deaths) | 347 |
| Number of covariates | 11 |
| **Events-per-variable (EPV)** | |
| **EPV ratio** | **31.55** |
