# Supplementary material for: TNM stage I pancreatic ductal adenocarcinoma patients without pathological risk may drive limited benefit from adjuvant chemotherapy
Source: Front Med (Lausanne). 2026 Sep 14;13:1914610. doi: 10.3389/fmed.2026.1914610 (PMC13618324; doi:10.3389/fmed.2026.1914610)
Supplement: Supplementary file 2 [file Table2.docx]

| Table S2. The reason for adjuvant chemotherapy omission | | |
| --- | --- | --- |
| **Reasons for Omission** | **Case(*n*)** | **Notes** |
| Poor postoperative recovery / Complications | 44 | Abdominal infection;  Grade B/C postoperative pancreatic fistula (POPF);  Postoperative hemorrhage;  Delayed gastric emptying (DGE) |
| Poor performance status / Comorbidities | 31 | Advanced age  Severe cardiac/pulmonary/hepatic/renal dysfunction |
| Patient preference / Refusal | 57 |  |
| Physician decision - deemed low risk | 14 | Stage T1 |
| Other reasons | 109 | Financial constraints;  Loss to follow-up;  Transfer to another hospital |
