# Supplementary material for: TNM stage I pancreatic ductal adenocarcinoma patients without pathological risk may drive limited benefit from adjuvant chemotherapy
Source: Front Med (Lausanne). 2026 Sep 14;13:1914610. doi: 10.3389/fmed.2026.1914610 (PMC13618324; doi:10.3389/fmed.2026.1914610)
Supplement: Supplementary file 3 [file Table3.docx]

| Table S3. Exploratory subgroup analysis of overall survival according to specific adjuvant chemotherapy regimens | | | |
| --- | --- | --- | --- |
| **Adjuvant Regimen** | **N** | **HR (95% CI)** | ***p*** |
| No Chemotherapy | 255 | 1(Ref) | - |
| GA | 33 | 0.514 (0.304-0.870) | 0.013 |
| GS | 75 | 0.524 (0.373-0.737) | ＜0.001 |
| GEMOX | 54 | 0.626 (0.445-0.880) | 0.007 |
| (m)FOLFIRINOX | 12 | 0.487 (0.200-1.184) | 0.024 |
| S-1 | 45 | 0.390 (0.246-0.618) | ＜0.001 |
| GEM | 45 | 0.613 (0.397-1.023) | 0.041 |
