# Supplementary material for: TNM stage I pancreatic ductal adenocarcinoma patients without pathological risk may drive limited benefit from adjuvant chemotherapy
Source: Front Med (Lausanne). 2026 Sep 14;13:1914610. doi: 10.3389/fmed.2026.1914610 (PMC13618324; doi:10.3389/fmed.2026.1914610)
Supplement: Supplementary file 4 [file Table4.docx]

| Table S4. The clinicopathological characteristics of the TNM stage I patients with pancreatic ductal adenocarcinoma | | | | |
| --- | --- | --- | --- | --- |
| **Characteristics** | **TNM stage I** | | ***x^2^*** | ***p*** |
|  | **PRFs(n=)** | **NRFs(n=)** |  |  |
| Gender |  |  | 1.033 | 0.309 |
| Male/female | 78/64 | 31/18 |  |  |
| Age at surgery ($\bar{x}\pm s$) | 60.57$\pm9.020$ | 59.76$\pm8.278$ |  | 0.578 |
| Tumor location |  |  | 1.835 | 0.177 |
| Head | 102 | 40 |  |  |
| Body or tail | 40 | 9 |  |  |
| 8th T stage |  |  | 3.997 | **0.046** |
| T1 | 34 | 19 |  |  |
| T2 | 108 | 30 |  |  |
| Preoperative CA19-9 (U/ml) |  |  | 0.875 | 0.646 |
| < 200 | 85 | 33 |  |  |
| 200 - 1000 | 42 | 12 |  |  |
| ≥ 1000 | 15 | 4 |  |  |
| Preoperative CEA (ng/ml) |  |  | 0.375 | 0.550 |
| ≤ 5 | 113 | 37 |  |  |
| > 5 | 29 | 12 |  |  |
| Perineural invasion |  |  | 7.432 | **0.006** |
| Absent | 47 | 27 |  |  |
| Present | 95 | 22 |  |  |
| Lymphovascular invasion |  |  | 1.330 | 0.249 |
| Absent | 125 | 46 |  |  |
| Present | 17 | 3 |  |  |
| Postoperative complications |  |  | 0.000 | 0.990 |
| Present | 32 | 11 |  |  |
| Absent | 110 | 38 |  |  |
| Postoperative adjuvant chemotherapy |  |  | 0.034 | 0.854 |
| No | 63 | 21 |  |  |
| Yes | 79 | 28 |  |  |
