# Supplementary material for: TNM stage I pancreatic ductal adenocarcinoma patients without pathological risk may drive limited benefit from adjuvant chemotherapy
Source: Front Med (Lausanne). 2026 Sep 14;13:1914610. doi: 10.3389/fmed.2026.1914610 (PMC13618324; doi:10.3389/fmed.2026.1914610)
Supplement: Supplementary file 5 [file Table5.docx]

| Table S5. Subgroup analysis of overall survival benefit associated with adjuvant chemotherapy | | | | |
| --- | --- | --- | --- | --- |
| Subgroup | Chemotherapy Group (n) | No Chemotherapy Group (n) | HR (95%CI) | *p* |
| **By pathological risk factors (PRFs)** | | | | |
| NRFs | 51 | 38 | 0.577 (0.309-1.077) | 0.021 |
| PRFs | 214 | 217 | 0.554 (0.442-0.694) | ＜0.001 |
| **By tumor stage (among NRF group)** | | | | |
| TNM stage I | 28 | 21 | 0.491 (0.186-1.299) | 0.365 |
| TNM stageⅡ | 19 | 14 | 0.668 (0.256-1.748) | 0.041 |
| TNM stageⅢ | 4 | 3 | 0.215 (0.069-2.101) | 0.047 |
