# Supplementary material for: TNM stage I pancreatic ductal adenocarcinoma patients without pathological risk may drive limited benefit from adjuvant chemotherapy
Source: Front Med (Lausanne). 2026 Sep 14;13:1914610. doi: 10.3389/fmed.2026.1914610 (PMC13618324; doi:10.3389/fmed.2026.1914610)
Supplement: Supplementary file 6 [file Table6.docx]

| Table S6. TNM stage Ⅰ/ NRFs of overall survival benefit associated with adjuvant chemotherapy | | | | |
| --- | --- | --- | --- | --- |
|  | Chemotherapy Group (n) | No Chemotherapy Group (n) | HR (95%CI) | *p* |
| OS | 28 | 21 | 1.695 (0.664-4.323) | 0.270 |
| RFS | 28 | 21 | 1.204 (0.367-1.734) | 0.644 |
